# Antiapoptotic BCL2 family proteins BCL-XL and MCL1 as factors predicting resistance against venetoclax plus azacitidine for patients with newly diagnosed acute myelogenous leukemia

**DOI:** 10.1371/journal.pone.0341461

**Published:** 2026-01-30

**Authors:** Yusuke Kamihara, Shohei Kikuchi, Nanako Ishikawa, Nozomi Shinomiya, Kohei Kunimoto, Ryusuke Horaguchi, Takuma Fujihira, Yoshimi Nabe, Tomoki Minemura, Kento Ono, Akinori Wada, Nam H. Dang, Tsutomu Sato

**Affiliations:** 1 Department of Hematology, Toyama University School of Medicine, Toyama, Toyama, Japan; 2 Division of Hematology/Oncology, University of Florida, Gainesville, Florida, United States of America; European Institute of Oncology, ITALY

## Abstract

The combination of Venetoclax (VEN), a BCL2 inhibitor, and Azacitidine (AZA), a hypomethylating agent, is the standard treatment for acute myelogenous leukemia (AML) in patients older than 65 years who are not eligible for intensive chemotherapy. While high response rates for this treatment have been noted, it has been also reported that the anti-apoptotic BCL2 family proteins BCL-XL and MCL1 may be involved in VEN resistance. However, no study has heretofore been conducted to investigate the effectiveness of treatment and the expression of BCL-XL or MCL1 in patients treated with VEN + AZA therapy. In this study, we analyzed blasts from patients with newly diagnosed AML treated with VEN + AZA therapy by qPCR, confirmed by siRNA in cultured cell lines, and evaluated the validity of the immunostaining method. We demonstrated that BCL-XL or MCL1 was highly expressed in leukemia cells of patients who did not respond to this treatment. In addition, leukemia cells from patients who had responded to VEN + AZA but relapsed during the course of treatment showed increased expression of BCL-XL or MCL1 compared to pre-treatment levels. Furthermore, downregulation of BCL-XL expression in a VEN-resistant AML cell line with siRNA increased sensitivity to VEN. On the other hand, the expression of BCL-XL and MCL1 in leukemia cells could be easily semi-quantified by immunostaining, with these results correlating with those obtained by qPCR. These results indicate that immunostaining for BCL-XL and MCL1 upon bone marrow examination at diagnosis not only can predict susceptibility to VEN + AZA therapy, but may also be useful for patient stratification for VEN + AZA treatment in the future.

## Introduction

Venetoclax (VEN) is a highly selective and effective inhibitor of B-cell/chronic lymphocytic leukemia lymphoma 2 (BCL2) that has revolutionized the treatment of hematologic malignancies, showing remarkable efficacy particularly in CLL [1–3]. The combination of VEN and azacitidine (AZA), a hypomethylating agent, has been established as an excellent therapeutic strategy for newly diagnosed AML patients, particularly those ineligible for standard remission induction therapy. Clinical trial results demonstrated the superiority of VEN + AZA over AZA plus placebo in terms of both overall survival and response rates [4]. However, given the fact that approximately one-third of patients (33.6%) did not achieve CR + CRi even with VEN + AZA [4], the management and optimization of therapeutic strategies for VEN + AZA resistance remains an important and urgent issue.

Analyzing clinical and cytogenetic data with the aim of extracting factors involved in resistance to VEN + AZA therapy, several studies have compared patient outcomes. These studies have identified various clinical and genetic variables (e.g., age, disease subtype, and *RUNX1* status) that influence response, although a reliable molecular biomarker for predicting VEN + AZA resistance remains elusive [5,6].

Meanwhile, several studies have attempted to examine VEN resistance based on the mechanism of action of VEN. VEN induces apoptosis in leukemic cells by inhibiting the anti-apoptotic protein BCL2. This inhibition results in the release of pro-apoptotic factors, such as BCL2 like 11 (BIM). However, resistance may occur because VEN does not target the other anti-apoptotic proteins, B-cell lymphoma-extra large (BCL-XL) and myeloid cell leukemia sequence 1 (MCL1). These proteins are capable of sequestering the released BIM and other pro-apoptotic BCL-2 family members, thereby maintaining cell survival and conferring resistance to VEN [7–9]. In this context, studies have examined VEN resistance by evaluating the balance of antiapoptotic and proapoptotic effects caused by these competing BCL-2 family proteins. Understanding these resistance mechanisms is crucial for developing strategies to overcome them, such as exploring novel therapeutics and biomarkers [10,11].

Several studies using cultured cell lines have been published to investigate inherent VEN resistance. These investigations consistently demonstrated the strong involvement of the anti-apoptotic proteins BCL-XL and MCL1. Specifically, Wang Q et al. [12] reported that high expression of BCL-XL was detected only in the highly VEN-resistant cell line U-937. Lin KH et al. [13] showed that forced expression of BCL-XL and MCL1 reduced VEN sensitivity in AML cell lines. Furthermore, Pan R et al. [14] demonstrated that BCL-XL expression levels quantified by Western blot positively correlated with VEN IC50 values, and functionally confirmed that manipulating the expression of MCL1 or BCL-XL (knockdown or forced expression) altered VEN sensitivity.

Evaluation of acquired resistance to VEN has also been undertaken. Multiple studies (Sharon D et al. [15], Lin KH et al. [13], and Konoplev SN [16]) generated VEN-resistant AML cell lines by long-term exposure to slowly escalating concentrations of VEN. These three studies consistently demonstrated that the upregulation of anti-apoptotic proteins, such as MCL1 (in MOLM-13) or BCL-XL (in MV4–11 and OCI-AML2), serves as a major mechanism when cells acquire VEN resistance.

In addition to cell line studies, patient specimens have also been used to examine issues regarding VEN resistance. A Phase II, single-arm study utilized BH3 profiling—a method that reveals dependence on BCL2 family proteins for survival in response to apoptotic stimuli induced by BH3 only proteins. This analysis was conducted using a limited number of 12 patient samples [17]. As a result, a negative correlation was observed between the dependence on BCL-XL and MCL1 for survival and the duration of VEN administration. These findings suggest that VEN treatment may not be successful if MCL1 or BCL-XL is inhibitory to BH3 only protein stimulation [8].

As described above, studies using cultured cells and patient specimens suggest that VEN resistance may be related to BCL-XL and MCL1. However, there have been no reports to date examining the expression of BCL-2, BIM, BCL-XL, and MCL1 in AML cells from patient samples and their response to VEN + AZA. In this paper, we initially evaluated expression levels by quantitative polymerase chain reaction (qPCR), and subsequently used immunostaining since the latter technique is more easily done in daily clinical practice. Findings from our present work suggest that treatment response can be predicted by examining BCL-XL and MCL1 expression, which may also be used in the future for potential stratification strategy.

## Materials and methods

### Patients

Previously untreated patients with confirmed AML who underwent chemotherapy with VEN + AZA at the Department of Hematology, University of Toyama Hospital between May 1, 2018, and December 31, 2022, were included. The Institutional Review Board of Toyama University Hospital approved this study on February 22, 2023 (protocol no. R2022198) and waived the requirement for written informed consent because only existing database records and residual clinical specimens were used. Instead, we employed an opt-out consent model: study objectives, eligibility criteria, and the types of data and samples to be used were posted on our institutional website, and patients could decline participation via e-mail, telephone, or fax. Clinical trial number: not applicable. The data were accessed retrospectively from March 2023 to February 2024. Although the authors had access to information that could identify individual participants during data collection, we replaced medical record numbers with unique codes and ensured that no identifiable information was included in the recorded data.

### Dosing schedule

The dosing schedule is as previously reported [4]. Namely, VEN was administered orally, once daily, with food. For mitigation of the tumor lysis syndrome during cycle 1, the dose of VEN was 100 mg on day 1 and 200 mg on day 2; on day 3, the target dose of 400 mg was reached and continued until day 28. In all subsequent 28-day cycles, the dose of VEN was initiated at 400 mg daily. Patients also received AZA at a dose of 75 mg per square meter of body-surface area, subcutaneously or intravenously, on days 1–7 every 28-day cycle. In some patients, the dosage of VEN and/or AZA after the second cycle was appropriately reduced based on the judgment of each attending physician.

### Best response

At the start of the second cycle, the best response was determined based on previous reports [18]. The details are as follows. CR**:** No morphologic evidence of AML and absolute neutrophil count ≥ 10^3^/µL (≥ 1.0 x 10^9^/L), platelets ≥ 10^5^/µL (≥100 x 10^9^/L), red cell transfusion independence, and bone marrow with < 5% blasts. Absence of circulating blasts and blasts with Auer rods; absence of extramedullary disease. CRi: All criteria as CR except for residual neutropenia < 10^3^/µL (1,000/µL) or thrombocytopenia < 10^5^/µL (100,000/µL). If all criteria for CR are met except for red blood cell transfusion independence, this also fulfills CRi criteria. Partial Remission (PR): All of the hematologic values for a CR but with a decrease of at least 50% in the percentage of blasts to 5% to 25% in the bone marrow aspirate. Resistant Disease (RD): Failure to achieve CR, CRi, PR; only for patients surviving at least 7 days following completion of Cycle 1 treatment, with evidence of persistent leukemia by blood and/or bone marrow examination. PD: 50% increase in marrow blasts over baseline (a minimum 15% point increase is required in cases with < 30% blasts at baseline; or persistent marrow blast percentage of > 70% over at least 3 months; without at least a 100% improvement in absolute neutrophil count to an absolute level (> 0.5 x 10^9^/L, and/or platelet count to > 50 x 10^9^/L non-transfused); or 50% increase in peripheral blasts (white blood cell x % blasts) to > 25 x 10^9^/L (> 25,000/µL); or new extramedullary disease.

### Clinical samples

Bone marrow mononuclear cells were isolated from bone marrow puncture fluid using Lymphoprep^TM^ (Serumwerk Bernburg AG, Bernburg, Germany). Leukemic cells were then further isolated using immunomagnetic beads, CD34 or CD33 MicroBead Kit^TM^ (Order no. 130-046-702 or 130-045-501, respectively) (Miltenyi Biotec, Auburn, CA, USA), according to the manufacturer’s instructions.

### Real-time quantitative reverse transcription PCR

Expression level of mRNA was estimated by qPCR method. Cells were lysed and total RNA was extracted using the RNeasy Plus Mini Kit (QIAGEN, Hilden, Germany) according to the manufacturer’s instructions. Total RNA (1 µg) was reverse transcribed using the QuantiTect Rev. Transcription Kit (QIAGEN). Quantification of mRNA was performed using the iQ5 Multicolor Real-Time PCR Detection System (Bio-Rad, Hercules, CA, USA) and the QuantiTect SYBR Green PCR Kit (QIAGEN). Obtained data were analyzed using iQ™5 Optical System Software, Version 2.1 (Bio-Rad), being normalized to vacuolar protein sorting-associated protein 33B (VPS33B) expression [19]. Relative gene quantitation was calculated by the 2^-delta Ct method. The PCR primers used in this study are based on a previous report [19] and shown in S1 Table.

### Cell culture

The Histiocytic lymphoma cell line, U-937; the acute monocytic leukemia cell lines, THP-1; and the myelodysplastic syndrome cell line, SKM-1, were supplied by Japanese Collection of Research Bioresources Cell Bank (JCRB). All these cell lines were maintained in RPMI 1640 (Gibco BRL, Tokyo, Japan) supplemented with 10% heat-inactivated fetal bovine serum (Sigma, St. Louis, MO, USA), 100 µg/mL streptomycin, and 100 U/mL penicillin.

### Reagents

Venetoclax (ABT-199), Bcl-2 inhibitor was purchased from abcam (Cambridge, MA, USA). Silencer^TM^ Select Pre-Designed Short interfering RNA (siRNA) (*BCL2L1*, s1920 and *MCL1*, s8583) and Silencer^TM^ Select Negative Control No. 1 siRNA were purchased from ThermoFisher (Bedford, MA, USA).

### Cellular cytotoxicity

Viable cell number was quantified using a Premix WST-1 Cell Proliferation Assay System (TaKaRa, Shiga, Japan) according to the manufacturer’s instructions. Level of cytotoxicity was also quantified by measuring the level of lactate dehydrogenase (LDH) released from damaged cells using a Cytotoxicity LDH Assay Kit-WST (Dojindo, Kumamoto, Japan) according to the manufacturer’s instructions.

### Transfection of siRNAs

THP-1 cells were transfected with siRNAs using Lipofectamine^TM^ RNAiMAX Transfection Reagent (ThermoFisher) according to the manufacturer’s instructions. Briefly, 2 x 10^5^ cells were transfected with 100 pmol siRNA using 3 µL transfection reagent mentioned above diluted with 100 µL Opti-MEM^TM^ I Reduced Serum Medium (ThermoFisher) and cultured for 48 hours. This gives a final siRNA concentration of 100 nM.

### Immunohistochemical staining

Expression of both BCL-XL and MCL1 in sections of bone marrow was assessed by immunohistochemistry using the standard protocol. In brief, following heat-induced antigen retrieval at 95°C in water bath with Target Retrieval Solution, High pH (Agilent Dako, Santa Clara, CA, USA) for 20 minutes, specimens of decalcified bone marrow core biopsy were incubated with monoclonal rabbit anti-human BCL-XL (54H6), #2764 or monoclonal rabbit anti-human MCL1 (D5V5L), #39224 (Cell Signaling Technology, Danvers, MA, USA) at dilution of 1:500 or 1:200, respectively for one hour at room temperature. For CD34 staining, following heat-induced antigen retrieval at 95°C for 30 minutes, samples were incubated with monoclonal mouse anti-human CD34 (QBEnd/10), #05267323001 (Roche, Rotkreuz, Switzerland) without dilution for 30 minutes at room temperature. Detection of immune complex on tissue section was done using Histofine Simple Stain MAX-PO (MULTI) (Nichirei Bioscience, Tokyo, Japan), which was visualized with DAB Substrate Kit (Nichirei Bioscience). A freely available software Fiji (Image J2, version 2.9.0) was used for quantitative analysis of protein expression [20,21].

### Statistical analysis

All statistical analyses were performed using GraphPad Prism 9 (GraphPad Software, La Jolla, CA). Statistical significance was determined using Welch’s t-test. Statistical significance was defined as *p* < 0.05.

## Results

### Patient characteristics

Information on the 12 patients analyzed in this study is shown in Table 1. The median age was 71 years (84–49), with eight males and four females. Based on the 2017 World Health Organization (WHO) classification, 5 patients had AML with myelodysplasia-related changes (MRC), 2 had therapy-related myeloid neoplasms, and one had acute monoblastic/monocytic leukemia. Four patients had AML with maturation, one of whom had post essential thrombocythemia (ET) and another had post primary myelofibrosis (PMF). For cytogenetics, 8 were complex, 3 were normal, and one was del(5q). Regarding *FLT3*-internal tandem duplication (*FLT3*-ITD) mutation, 5 were negative, 1 was positive, and 6 were not defined (ND). As for the Janus kinase 2 (*JAK2*) V617F mutation, two patients with post-ET and post-PMF were positive, and the other 10 were ND. Concerning Best Response, there were 0 CR, 5 CRi, 1 PR, 6 RD, and 0 PD. The source of leukemia cells used in the analysis was bone marrow (BM) in 11 patients and peripheral blood (PB) in one post-PMF patient. The median percentage of blasts in the BM was 44% (22–78). The markers used to select leukemic cells were CD34 in 8 patients and CD33 in 4 patients.

**Table 1:** AML patient characteristics.

UPN	Age	Sex	WHO classification (2017)	Cytogenetics	FLT3-ITD	JAK2 V617F	Best Response	Source	% Blasts in BM	Selection marker
1	68	F	AML-MRC	Normal	Negative	ND	CRi	BM	47	CD33
2	71	M	AML with maturation	Del (5q)	Negative	ND	CRi	BM	27	CD33
3	71	M	AML with maturation (post ET)	Normal	ND	Positive	CRi	BM	62	CD34
4	82	M	AML-MRC	Complex	Negative	ND	CRi	BM	43	CD33
5	80	F	AML with maturation	Complex	Negative	ND	CRi	BM	57	CD34
6	75	F	Acute monoblastic/monocytic leukemia	Normal	ND	ND	PR	BM	78	CD33
7	71	M	AML-MRC	Complex	ND	ND	RD	BM	48	CD34
8	84	M	Therapy-related myeloid neoplasms	Complex	ND	ND	RD	BM	44	CD34
9	73	M	AML-MRC	Complex	Positive	ND	RD	BM	23	CD34
10	49	M	Therapy-related myeloid neoplasms	Complex	ND	ND	RD	BM	30	CD34
11	55	F	AML with maturation (post PMF)	Complex	Negative	Positive	RD	PB	ND	CD34
12	66	M	AML-MRC	Complex	ND	ND	RD	BM	22	CD34

AML, acute myeloid leukemia; UPN, unique patient number; F, female; M, male; WHO, world health organization; MRC, myelodysplasia-related changes; ET, essential thrombocythemia; PMF, primary myelofibrosis; CRi, complete remission with incomplete blood count recovery; PR, partial remission; RD, resistant disease; BM, bone marrow; PB, peripheral blood; ND, not defined.

### BCL-XL/MCL1 expression and VEN + AZA sensitivity

**Fig 1:**
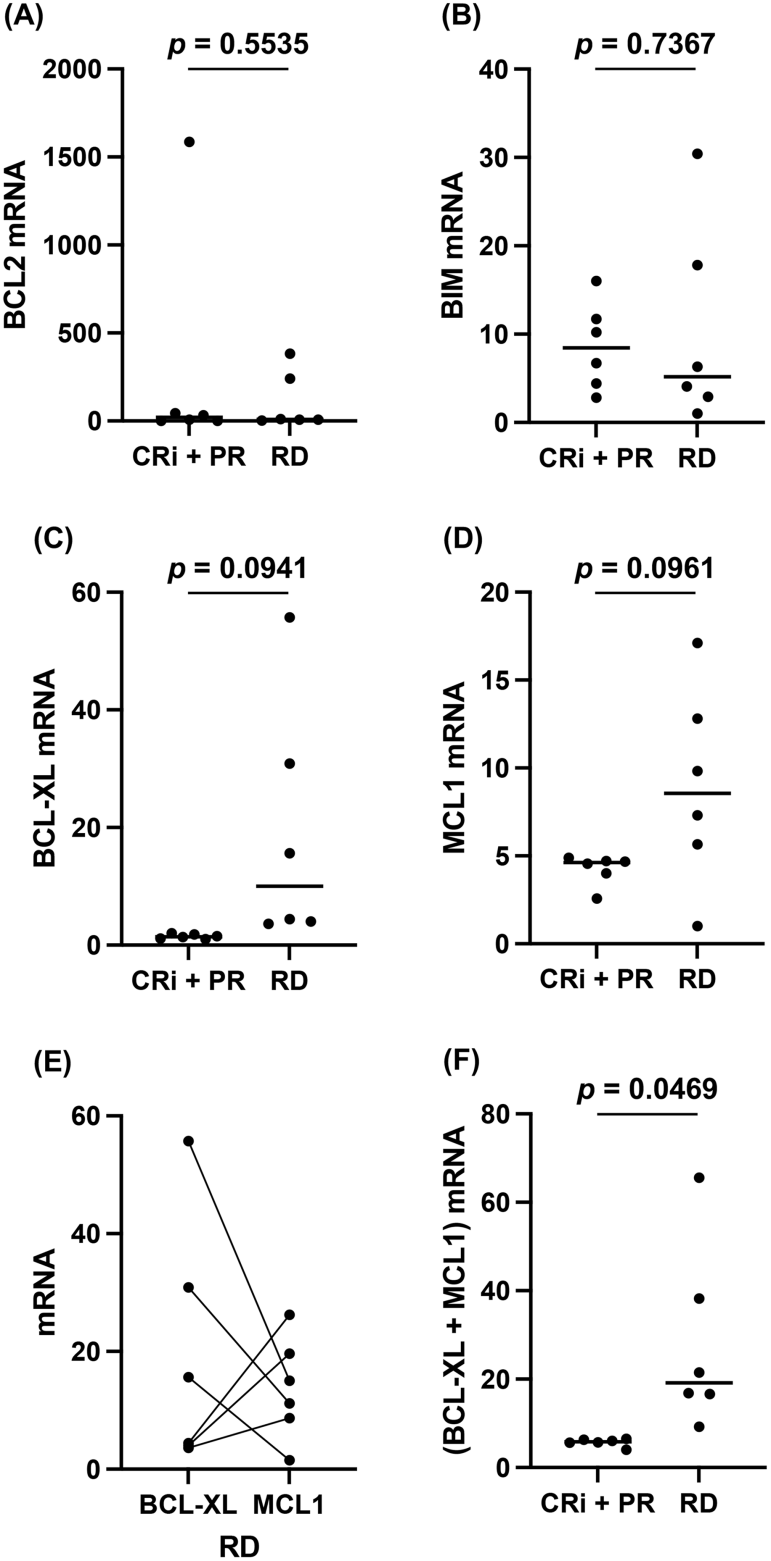
Expression levels of BCL2, BIM, BCL-XL, and MCL1 mRNA in patients’ leukemic cells at diagnosis. Expression levels of mRNA in patients’ leukemic cells were estimated by qPCR methods (n = 3). **(A)** BCL2, **(B)** BIM, **(C)** BCL-XL, and **(D)** MCL1 were compared between patients with CRi + PR and those with PD. **(E)** BCL-XL and MCL1 were compared in patients with RD. **(F)** BCL-XL + MCL1 were compared between patients with CRi + PR and those with PD.

We examined the expression of BCL2, BIM, BCL-XL, and MCL1 in patient leukemia cells at diagnosis and before treatment (Fig 1). Comparing the 6 CRi + PR to the 6 RD patients, no significant differences were found in BCL2 and BIM (*p* = 0.5535 or *p* = 0.7367, respectively) (Fig 1A-B). Compared to these *p*-values, although much smaller, the *p*-values for BCL-XL and MCL1 were also not less than 0.05 (*p* = 0.0941 or *p* = 0.0961, respectively) (Fig 1C-D). However, when comparing BCL-XL and MCL1 for the six RD patients, we found that if one of them was high, the other was low (Fig 1E). Together, the sum of BCL-XL and MCL1 was higher in the RD cohort than CRi + PR (*p* = 0.0469) (Fig 1F). These results indicate that BCL-XL and/or MCL1 expression may be involved with resistance to initial treatment of VEN + AZA.

**Fig 2:**
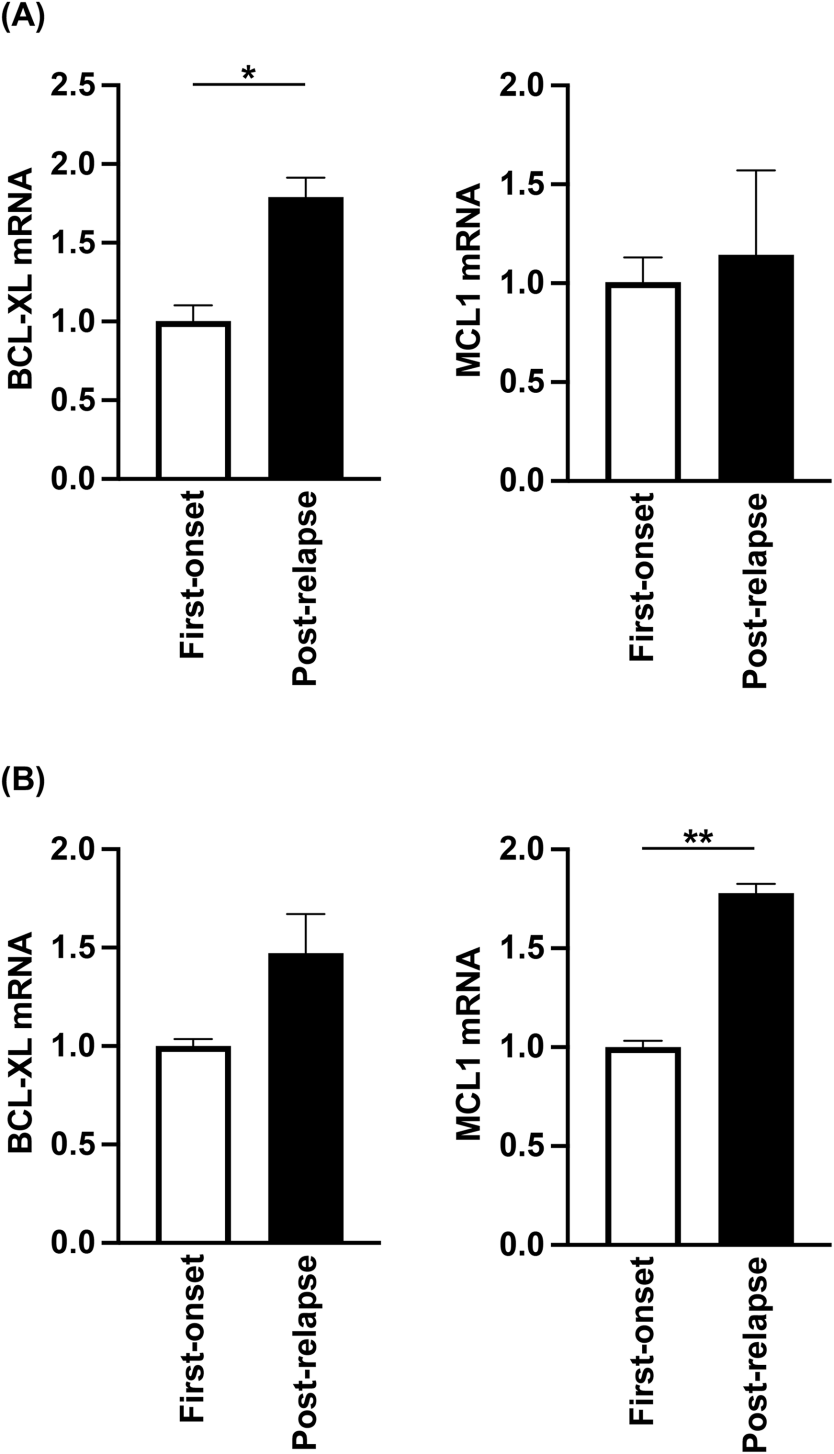
Expression levels of BCL-XL and MCL1 mRNA in patients’ leukemic cells post-relapse. Expression levels of mRNA in patients’ leukemic cells were estimated by qPCR methods (n = 3). BCL-XL and MCL1 were compared between newly diagnosed and post-relapse patient samples. **(A)** UPN5, and **(B)** UPN6. Data are shown as mean ± SD. * *p* < 0.05, ** *p* < 0.001.

We next examined the leukemia cells at relapse. Specifically, although UPN5 and UPN6 acquired CRi after the first cycle, UPN5 was determined to be PD after the 13th cycle and UPN6 after the 5th cycle. Comparing leukemia cells at relapse with those obtained at diagnosis prior to treatment, BCL-XL was elevated in UPN5 (Fig 2A) (*p* < 0.05), and MCL1 was enhanced in UPN6 (Fig 2B) (*p* < 0.001). These results suggest that increased expression of BCL-XL and/or MCL1 may also be involved in acquired resistance.

### Increased VEN sensitivity associated with decreased BCL-XL expression

**Fig 3:**
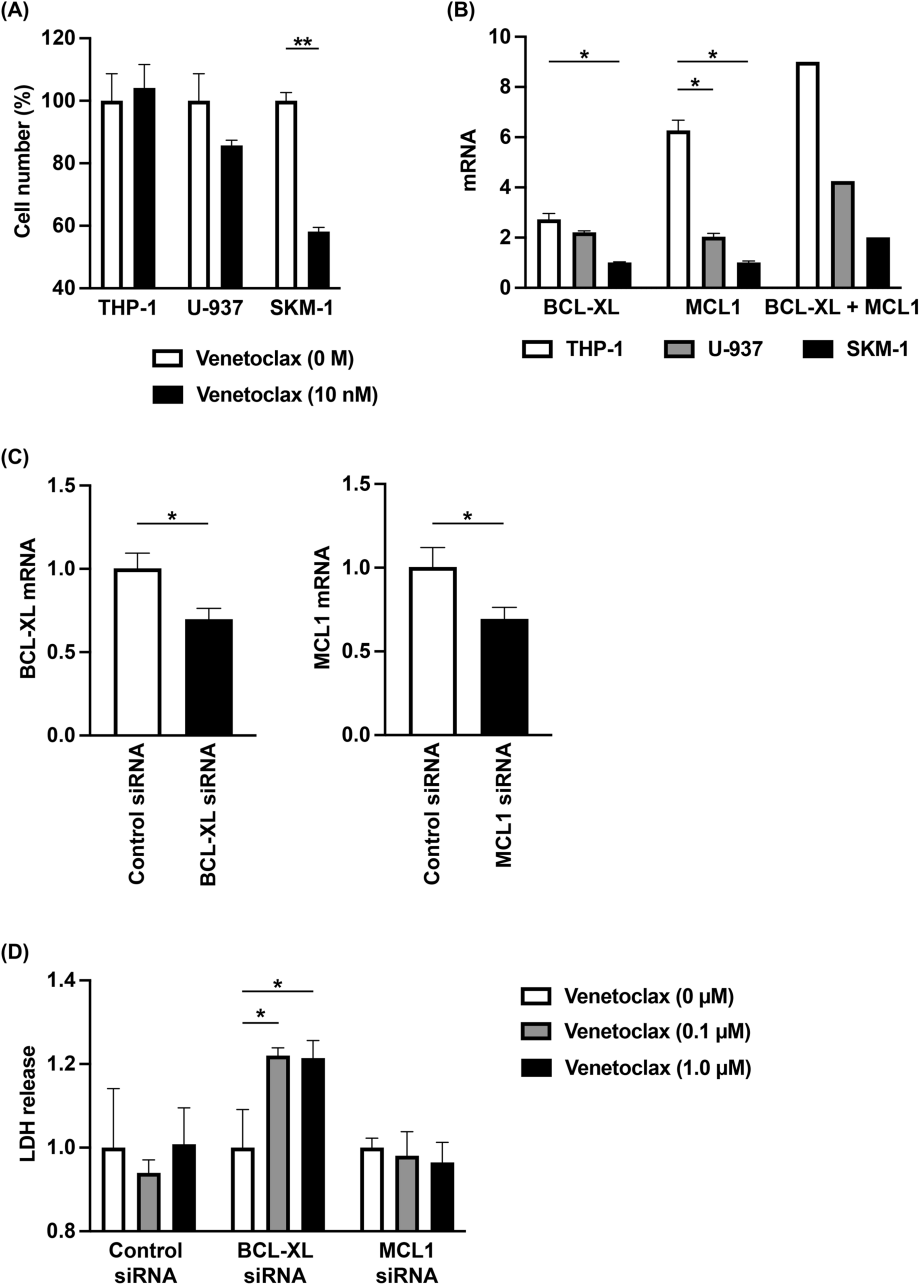
Effects of down-regulated BCL-XL mRNA on VEN susceptibility. (**A**) 1 x 10^5^ of hematological cancer cell lines (THP-1, U-937, or SKM-1) were cultured with venetoclax (0 or 10 nM) for 72 **h.** Cell number was estimated by a colorimetric assay using WST-1 reagent (n = 3). **(B)** Expression levels of mRNA (BCL-XL and MCL1) in hematological cancer cell lines (THP-1, U-937, or SKM-1) were estimated by qPCR methods (n = 3). BCL-XL + MCL1 were compared between three cell lines. **(C)** Expression levels of mRNA (BCL-XL and MCL1) in THP-1 cells treated with siRNA (control, BCL-XL, or MCL1) were estimated by qPCR methods (n = 3). **(D)** THP-1 cells treated with siRNA (control, BCL-XL, or MCL1) were cultured with venetoclax (0, 0.1, or 1.0 µM) for 6 **h.** Cytotoxicity was estimated by a LDH release assay (n = 6). Data are shown as mean ± SD. * *p* < 0.05, ** *p* < 0.001.

We next performed studies using three hematological cancer cell lines, THP-1, U-937, and SKM-1. Experiments evaluating viable cell counts with the addition of VEN showed that THP-1 was VEN-resistant compared to SKM-1 (Fig 3A). In addition, expression of BCL-XL and MCL1 in THP-1 were both higher than in SKM-1 (Fig 3B). To investigate whether VEN resistance of THP-1 is dependent on high expression of BCL-XL and MCL1, siRNA-based knockdown of BCL-XL and MCL1 was performed, which effectively suppressed both mRNA expressions (Fig 3C). As a result, VEN sensitivity was enhanced with the suppression of BCL-XL expression. In contrast, suppression of MCL-1 expression did not alter VEN sensitivity (Fig 3D).

### Suitability of ICH method in predicting VEN + AZA susceptibility

**Fig 4:**
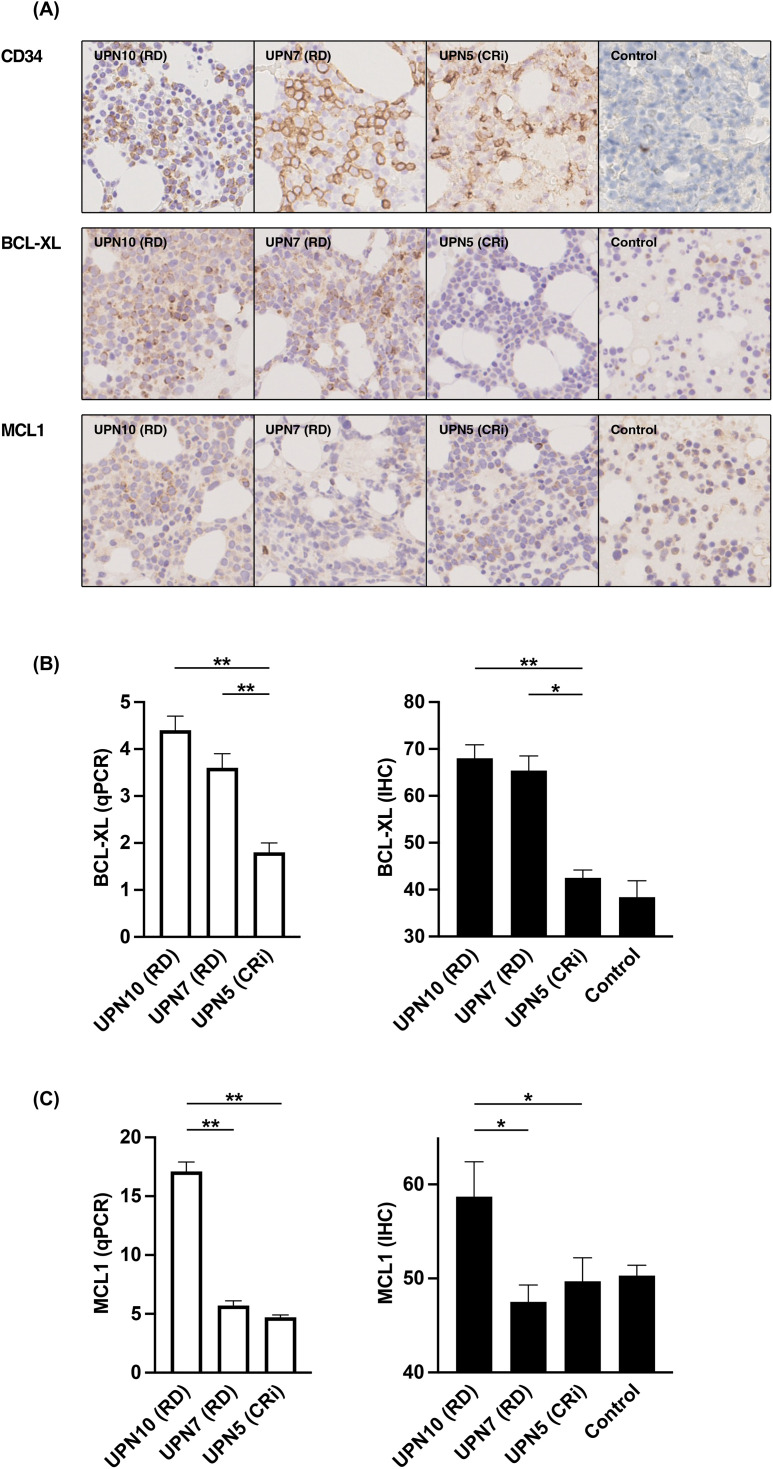
Quantitative analysis of BCL-XL and MCL1 using immunohistochemical method. **(A)** Immunostaining results of BCL-XL and MCL1. Control staining is CD34. Samples are bone marrow core biopsy of both UPN10 and UPN7 with RD, and UPN5 with CRi. Control sample is that of lymphoma patient without bone marrow involvement. **(B and C)** Quantitative analysis of mRNA(qPCR) (left; white columns) and protein (IHC) (right; black columns) expression of BCL-XL (**B**) and MCL1 **(C)**. Samples are the same as **(A)** (n = 3). Data are shown as mean ± SD. * *p* < 0.05, ** *p* < 0.001.

Immunohistochemistry (IHC) is technically easier to perform in routine clinical practice and is therefore more practical than qPCR as a tool to analyze BCL-XL and MCL1 protein expression to predict response to VEN + AZA. Therefore, we examined whether IHC data could be a substitute for data obtained from qPCR (Fig 4). IHC analysis of BCL-XL and MCL1 expression from UPN10 and UPN7, which were both RD, and UPN5, which was CRi, are shown in Fig 4A. Additionally, we quantified the expression of BCL-XL from IHC assay and compared it to mRNA expression in qPCR, as shown in Fig 1 (Fig 4B). Expression levels from UPN10 and UPN7 were higher than that from UPN5 in qPCR assay, and this difference could be reproduced in IHC analysis. Further results for MCL1 are shown in Fig 4C. Expression level from UPN10 was higher than that from UPN7 and UPN5 in the qPCR assay, and this difference was also reproducible in IHC analysis. Our findings indicate that VEN + AZA susceptibility can be predicted by IHC analysis alone, without the need for qPCR assay.

## Discussion

The availability of VEN, a selective inhibitor of BCL-2, has increased treatment options for AML patients. VEN in combination with AZA or low-dose cytarabine (LDAC) has become the standard treatment for newly diagnosed AML patients who are unable to receive intensive chemotherapy or are 75 years of age or older. With the emergence of VEN, there have been some reports that the current risk stratification methods for AML, such as the National Comprehensive Cancer Network (NCCN) and European LeukemiaNet (ELN), which are based on chromosomal abnormalities and genetic mutations, do not always match the actual real-world prognosis.

Miyashita N et al. [22] reviewed 89 AML patients who received initial treatment based on VEN and reported that the NCCN 2017 risk stratification did not match with overall survival. Meanwhile, they found that mutations such as *DNMT3A*, *IDH1*, *IDH2*, and *NPM1* are VEN high-susceptibility factors, while complex karyotype (CK) and *TP53* mutations are VEN low-susceptibility factors. The study also found that risk assessment using these factors clearly stratifies overall survival.

Döhner H et al. [23] classified AML patients enrolled in an open-label, non-randomized phase 1b trial (NCT02203773) and the confirmatory VIALE-A phase 3 randomized trial (NCT02993523), and treated with VEN + AZA according to the ELN 2017 risk stratification. As a result, they reported that there was no difference in median survival between the good and intermediate prognosis groups. However, compared to these two groups, the median survival of the poor prognosis group was distinctly shorter, and patients with *TP53* and *RUNX1* mutation in particular had a clearly poorer prognosis.

Therefore, with the advent of VEN, the risk classification method for AML patients needs revision, and it may be necessary to include not only a review of chromosomal aberrations and genetic mutations, but also the expression levels of alternative anti-apoptotic BCL-2 family proteins such as BCL-XL and MCL1 as prognostic factors. Such a necessity has been demonstrated not only by our present work, but also by several previous studies [8,12–16]. Expression levels of BCL-XL and MCL1 may not only be useful for AML risk classification but may also be important for treatment stratification. Specifically, this novel treatment stratification approach may be used to guide alternative therapies for patients predicted to be VEN + AZA resistant, with BCL-XL and MCL1 being potential novel targets. Regarding this point, our work demonstrated that suppression of BCL-XL expression using shRNA increased VEN susceptibility (Fig 3D).

There have been several reports on alternative therapies targeting BCL-XL and MCL1 using cultured cell lines. Wang Q et al. [12] examined OCI-AML3 and U-937 cells, which are not sensitive to VEN, and showed that the MCL1-selective inhibitor A-1210477 was effective in the former and the BCL-XL-selective inhibitor A-1155463 was effective in the latter. Their analysis indicated that the VEN-resistant U-937 cells expressed high levels of BCL-XL, and THP-1 cells were VEN-sensitive, expressing low levels of BCL-XL. However, since THP-1 cells were VEN-resistant and expressed high levels of BCL-XL in our study (Fig 3A-B), we used siRNA to target THP-1 cells (Fig 3C).

The effects of A-1155463, a selective BCL-XL inhibitor, have also been evaluated in multiple myeloma, malignant lymphoma, and acute leukemia. Punnoose EA et al. [19] used qPCR method to analyze 21 multiple myeloma cell lines and reported that VEN sensitivity was inversely proportional to BCL-XL expression, which was directly proportional to the ratio of BCL2:BCL-XL. Furthermore, by Western blot analysis, they showed that VEN resistance correlated with BCL-XL protein levels, and demonstrated the efficacy of A-1155463 against VEN-resistant cell lines. Dolnikova A et al. [24] showed that there was a strong *in vitro* synergy between VEN and A-1155463 in combination, mainly in cultured cell lines such as lymphoma and acute leukemia, but also in patient samples. In addition, the association between BCL-XL expression and VEN resistance has been elucidated using knockout and forced expression techniques. Furthermore, the usefulness of the combination therapy was confirmed using an experimental mouse model.

Meanwhile, Lin KH et al. [13] prepared resistant cell lines by long-term exposure of OCI-AML2 cells to VEN and then showed that this acquired resistance was reversed by the combination of the BCL-XL inhibitor WEHI-539 and shRNA knockdown against MCL1.

Analyses involving samples from patients with AML have also been published. Moujalled DM et al. [25] reported on a combination with the MCL1 inhibitor S63845 and the BCL-2 inhibitor S55746. In an *in vitro* study using primary AML samples, this combination exhibited synergistic apoptosis- inducing activity. Moreover, in a mouse xenograft model using MV4–11 and OCI-AML3, this combination similarly showed synergistic effects on survival, while also significantly reducing the number of primary AML samples grown in the bone marrow of mice. Ramsey HE et al. [26] reported on the effect of the combination of VU661013, a novel selective MCL1 inhibitor, and VEN. Besides having *in vitro* synergistic effects on primary AML patient samples, this combination was also effective on samples from AML patients whose disease had relapsed and/or was refractory following VEN and LDAC therapy. Furthermore, this combination displayed synergistic effect in an *in vivo* experimental system of mice implanted with patient samples.

For AML patients with high BCL-XL/MCL1 expression, it is possible that their disease may be resistant to VEN treatment, based on our findings. For this population, alternative treatments may be considered, including such therapy as liposomal daunorubicin and cytarabine (CPX-351). CPX-351 is a liposomal formulation of cytarabine and daunorubicin fixed in a 5:1 molar ratio [27]. The efficacy of this agent was compared to cytarabine plus daunorubicin chemotherapy (7 + 3 regimen) in 309 older patients with newly diagnosed high-risk/secondary AML [28]. This study found that patients who benefited from CPX-351 were those with wild-type *FLT3*, treatment-related AML, AML with antecedent myelodysplastic syndrome (MDS) or chronic myelomonocytic leukemia (CMML), and those with a favorable/intermediate cytogenetic risk classification. So far, there is little evidence on whether CPX-351 can overcome VEN resistance, but the report of Bataller A et al. encouraged us [29]. Namely, they evaluated the combination of CPX-351 and Ven in 33 patients with relapsed/refractory AML. And 19 of them had been previously treated with Ven, but 37% (7/19) achieved CR + CRi with CPX-351.

## Conclusion

In this study, we examined newly diagnosed AML patients treated with VEN + AZA and showed by qPCR method that BCL-XL or MCL1 was highly expressed in leukemia cells of patients who did not respond to this treatment. In addition, leukemia cells from patients who had responded to VEN + AZA but relapsed during the course of treatment showed increased expression of BCL-XL and MCL1 compared to pre-treatment levels. Furthermore, we showed that siRNA downregulation of BCL-XL expression in VEN-resistant AML cell lines increased their sensitivity to VEN. Notably, we found that the expression of BCL-XL and MCL1 in leukemic cells could be easily semi-quantified by immunostaining, and the results correlated with those of qPCR assays. These results suggest the possibility that immunostaining of leukemic cells at the time of diagnosis for BCL-XL and MCL1 may allow for accurate prediction of susceptibility to VEN + AZA therapy, while also being useful for treatment stratification in the future.

We note that there are limitations to our current study, primarily stemming from the patient cohort. First and foremost, the number of patients analyzed is relatively small, and this constraint is compounded by the inherent biological and clinical heterogeneity of the cohort, which includes various disease subtypes (e.g., AML-MRC, therapy-related AML, and secondary AML). This limited sample size precluded a statistically powered analysis to rigorously account for all potential confounding clinical and genetic variables through multivariate modeling. Consequently, we were unable to perform detailed stratification to determine if the BCL-XL/MCL1 association is enriched in specific subtypes or to generate a statistically robust correlation coefficient (e.g., Pearson/Spearman r values) for IHC and qPCR data across all evaluable samples. Therefore, our findings serve as preliminary clinical corroboration rather than definitive evidence, and they are not yet sufficient to recommend routine BCL-XL/MCL1 testing in clinical practice. A large-scale, prospective study involving a greater number of patients and comprehensive multivariate analysis is essential to validate our findings and dissect the influence of patient heterogeneity. In addition, while we used siRNA as a method to suppress gene expression, a complete knockdown or knockout approach would likely have resulted in stronger, more definitive inhibition, potentially yielding clearer functional results in several cell lines.

## Supporting information

S1 Table The PCR primers. (DOCX)
