# Supplementary material for: Antiapoptotic BCL2 family proteins BCL-XL and MCL1 as factors predicting resistance against venetoclax plus azacitidine for patients with newly diagnosed acute myelogenous leukemia
Source: PLoS One. 2026 Jan 30;21(1):e0341461. doi: 10.1371/journal.pone.0341461 (PMC12857986; doi:10.1371/journal.pone.0341461)
Supplement: S1 Table — (DOCX) [file pone.0341461.s001.docx]

Supplementary Table 1. The PCR primers.

| Genes | Primers | |
| --- | --- | --- |
|  | Forward | Reverse |
| BCL2 | 5’-CCT GTG GAT GAC TGA GTA CCT GAA-3’ | 5’-GGG CCG TAC AGT TCC ACA AA-3’ |
| BIM | 5’-TGC CAG GCC TTC AAC CA-3’ | 5’-GTT CAG CCT GCC TCA TGG A-3’ |
| BCL-XL | 5’-AAT GAC CAC CTA GAG CCT TGG A-3’ | 5’-CTC GGC TGC TGC ATT GTT C-3’ |
| MCL1 | 5’-CGC TGG AGA CCT TAC GAC G-3’ | 5’-TTG ATG TCC AGT TTC CGA AGC-3’ |
| VPS33B | 5’-GAG ATC TGC CTC AAT GAA TAA ATC C-3’ | 5’-TGG AGC AGC TTC CT-3’ |
